# R3D-BLAST2: an improved search tool for similar RNA 3D substructures

**DOI:** 10.1186/s12859-017-1956-6

**Published:** 2017-12-28

**Authors:** Ching-Yu Yen, Jian-Cheng Lin, Kun-Tze Chen, Chin Lung Lu

**Affiliations:** Department of Computer Science, National Tsing Hua University, Hsinchu, 30013 Taiwan

**Keywords:** Computational biology, RNA, Tertiary structure, BLAST-like search tool, Structural alphabet

## Abstract

**Background:**

RNA molecules have been known to play a variety of significant roles in cells. In principle, the functions of RNAs are largely determined by their three-dimensional (3D) structures. As more and more RNA 3D structures are available in the Protein Data Bank (PDB), a bioinformatics tool, which is able to rapidly and accurately search the PDB database for similar RNA 3D structures or substructures, is helpful to understand the structural and functional relationships of RNAs.

**Results:**

Since its first release in 2011, R3D-BLAST has become a useful tool for searching the PDB database for similar RNA 3D structures and substructures. It was implemented by a structural-alphabet (SA)-based method, which utilizes an SA with 23 structural letters to encode RNA 3D structures into one-dimensional (1D) structural sequences and applies BLAST to the resulting structural sequences for searching similar substructures of RNAs. In this study, we have upgraded R3D-BLAST to develop a new web server named R3D-BLAST2 based on a higher quality SA newly constructed from a representative and sufficiently non-redundant list of RNA 3D structures. In addition, we have modified the kernel program in R3D-BLAST2 so that it can accept an RNA structure in the mmCIF format as an input. The results of our experiments on a benchmark dataset have demonstrated that R3D-BLAST2 indeed performs very well in comparison to its earlier version R3D-BLAST and other similar tools RNA FRABASE, FASTR3D and RAG-3D by searching a larger number of RNA 3D substructures resembling those of the input RNA.

**Conclusions:**

R3D-BLAST2 is a valuable BLAST-like search tool that can more accurately scan the PDB database for similar RNA 3D substructures. It is publicly available at http://genome.cs.nthu.edu.tw/R3D-BLAST2/.

## Background

Besides being involved in protein synthesis, RNAs have been found to perform other diverse functions in the cell, such as processing and modification of RNAs, regulation of gene expression, and degradation and translocation of proteins [1]. In principle, it is widely believed that the functions of RNAs are largely determined by their three-dimensional (3D) structures. In the past few years, both the number and the size of experimentally solved RNA 3D structures in the Protein Data Bank (PDB) [2] and Nucleic Acid Database (NDB) [3] have dramatically increased. Therefore, automatic software tools capable of rapidly and accurately searching the PDB database for similar RNA 3D structures or substructures are helpful for the annotation of RNA structures and functions. Since computing similarity between two RNA 3D structures is an intractable task [4], currently existing tools, including RNA FRABASE [5, 6], FASTR3D [7], R3D-BLAST [8] and RAG-3D [9], all employ some heuristic approaches to scan the PDB database for similar RNA 3D structures and/or substructures.

In principle, both RNA FRABASE and FASTR3D use pattern-based approaches to search the PDB database for RNAs that have exactly the same secondary (2D) structure as that of the query RNA. As for R3D-BLAST, it reduces RNA 3D structures into one-dimensional (1D) structural sequences according to some local structure features in the nucleotide backbone conformation and applies BLAST to the resulting 1D structural sequences for searching similar RNA 3D substructures. RAG-3D exploits a coarse-grained graph representation to describe RNA 3D structures as simplified 3D graphs and searches for RNA 3D substructures with the same graph topology (i.e. pattern of vertex connectivity) as the query RNA substructure.

The above method we used to implement R3D-BLAST [8] is the so-called structural alphabet (SA)-based method, which utilizes an SA with 23 structural letters to encode RNA 3D structures from the PDB database into 1D sequences of structural letters and continues to apply BLAST [10], a popular bioinformatics tool to find homologous nucleotide or amino acid sequences just according to their sequence similarity, to search the SA-encoded sequences for similar RNA 3D substructures. In fact, the search performance of R3D-BLAST largely depends on the capability of the SA letters for representing the most common backbone conformations of RNA nucleotides. As reported in [11], two pseudo-torsion angles (i.e. *η* and *θ*), which are dihedral angles defined based on C4 ^′^ and P atoms from consecutive bases, are adequate to represent the backbone conformation of an RNA nucleotide. Therefore, the SA mentioned above was previously constructed from a collection of 117 RNA 3D structures (with 9527 nucleotides in total) using the *η* and *θ* values of their nucleotide backbones. Since the public release of R3D-BLAST in 2011, however, several hundreds of new RNA 3D structures have been experimentally determined and also deposited in the PDB database. Therefore, it can be expected that these newly determined RNA 3D structures should allow us to construct a new and sufficiently high-quality SA that can be used to further improve the search performance of R3D-BLAST.

Another reason to upgrade our R3D-BLAST is that the PDB data files used by R3D-BLAST to retrieve their RNA 3D structures or uploaded by the user to run R3D-BLAST were in the PDB format only. However, the PDB format now is a legacy format, because the size of a structure represented in a single PDB formatted file was limited to 99,999 atoms and the relationships among their data items were implicit [12]. The mmCIF (macromolecular Crystallographic Information File) format released in 1997 does not have the limitations of the PDB format described above [13]. Therefore, the PDB entries have been mainly distributed in the mmCIF format since it became the standard format of PDB archive distribution in 2014.

In this study, we have upgraded our RNA structural search tool R3D-BLAST to develop a new web server named R3D-BLAST2 (meaning R3D-BLAST version 2) based on a totally new SA that is constructed from a representative and sufficiently non-redundant list of 876 atomic-resolution RNA 3D structures (with 65,154 nucleotides in total). In addition, we have modified the kernel program in R3D-BLAST2 so that it can retrieve RNA 3D structures from the PDB data files in the mmCIF format and also allows the user to upload an mmCIF formatted file to search for similar RNA 3D substructures. For validation, we have used a benchmark dataset of RNA 3D structures to test R3D-BLAST2 and compare its search performance with its previous version R3D-BLAST and other similar RNA structural search tools, such as RNA FRABASE, FASTR3D and RAG-3D. Our experimental results have finally shown that R3D-BLAST2 indeed outperforms R3D-BLAST, as well as RNA FRABASE, FASTR3D and RAG-3D, by searching a larger number of RNA 3D substructures resembling those of the query RNA.

## Methods

### Algorithm of R3D-BLAST2

**Fig. 1:**
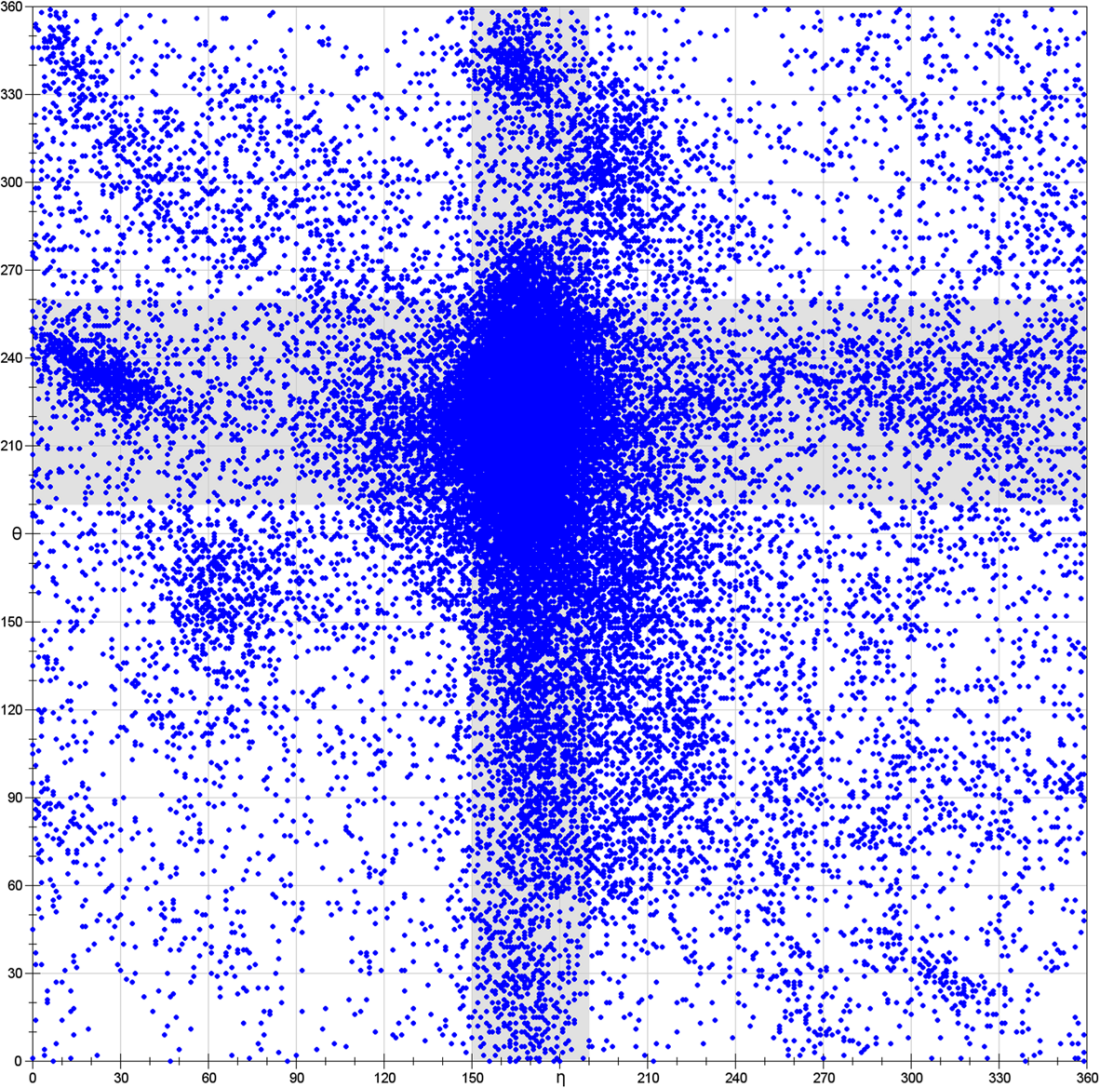
An *η*- *θ* plot of all non-terminal nucleotides from a non-redundant list of 876 RNA 3D structures, where the intersection of perpendicular gray regions (150° ≤*η*≤ 190° and 190° ≤*θ*≤ 260°) is designated the helical region

**Fig. 2:**
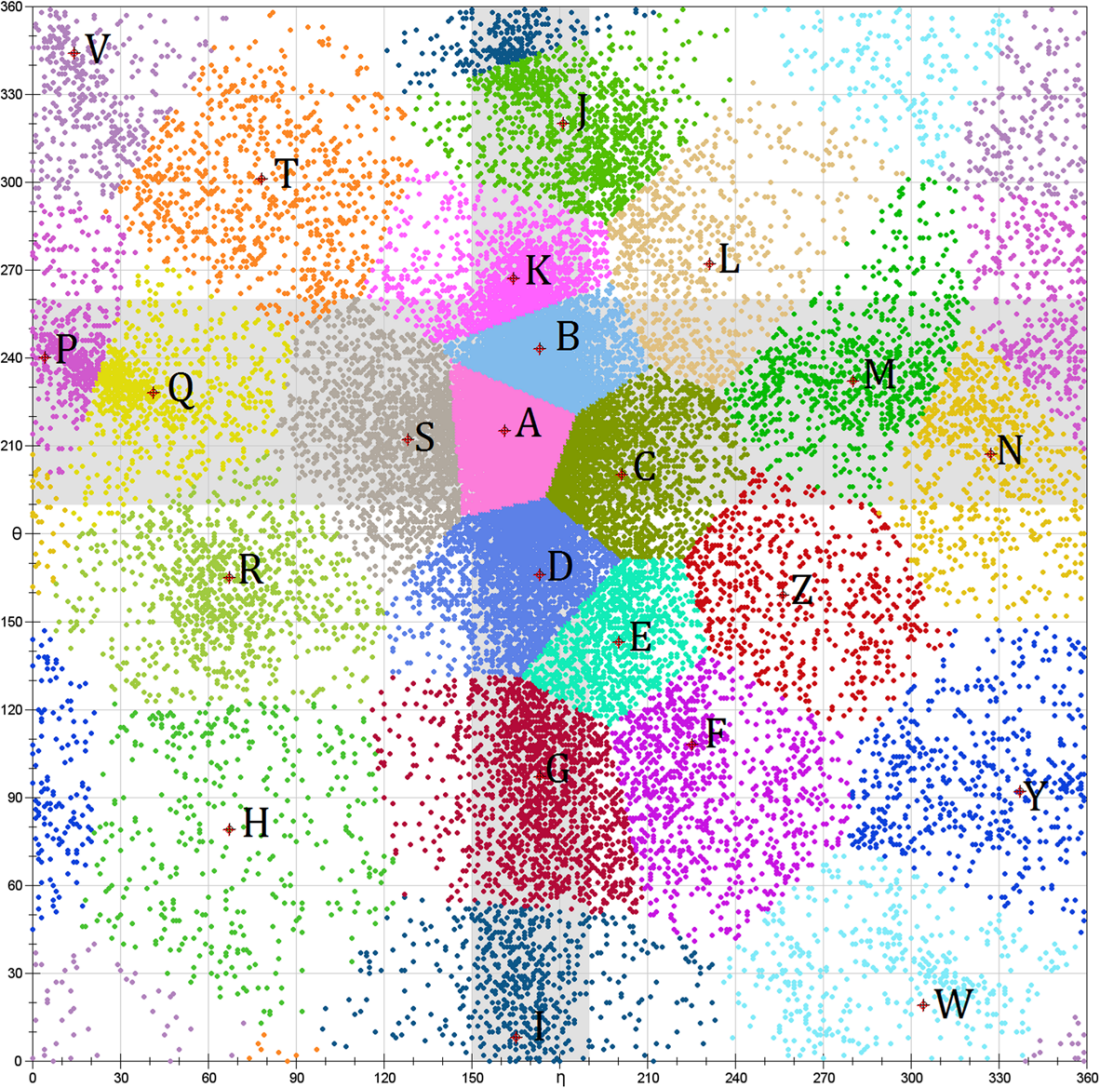
Twenty-three clusters classified by the AP algorithm based on pseudo-torsion angles of RNA backbone conformations

We used the following SA-based algorithm to implement R3D-BLAST2. First, we collected 63 402 non-terminal nucleotides (see Fig. 1 for their *η*- *θ* plot) from a representative and sufficiently non-redundant list of 876 RNA 3D structures determined by X-ray crystallography and electron microscopy (resolution ≤4 Å) from the RNA 3D Hub [14] and applied the affinity propagation (AP) algorithm [15] to classify these nucleotides into 23 conformation clusters (see Fig. 2) using their *η* and *θ* values. In principle, the AP algorithm is a message-passing method that operates by simultaneously considering all nucleotides as possible exemplars (i.e. centers) and recursively exchanging two types of real-valued messages, called responsibility and availability, between nucleotides until a collection of suitable exemplars and corresponding clusters appears. The *responsibility*, denoted by *r*(*i,k*), is sent from nucleotide *i* to candidate exemplar *k* and reflects the accumulated evidence for how well *k* can serve as the exemplar for *i* with taking into account other potential exemplars. The *availability*, denoted by *a*(*i,k*), is sent from candidate exemplar *k* to nucleotide *i* and reflects the accumulated evidence for how appropriate it would be for *i* to select *k* as its exemplar with taking into account the support from other nucleotides that *k* should be an exemplar. Note that the AP algorithm has been shown to perform better than *K*-centers clustering algorithm, which tends to fall into local optimality and is susceptive to noise and outliers [15]. In fact, nucleotides in each cluster usually are structurally similar in backbone geometry and hence we can use the center nucleotide to represent all the nucleotides in the cluster. For reducing RNA 3D structures into 1D structural sequences, we chose a capital letter to represent each of the 23 clusters as named in Table 1. Next, we utilized the collection of the 23 capital letters to form a structural alphabet and transformed all RNA 3D structures currently deposited in the PDB database (as of 23 March 2017) into 1D SA-encoded sequences based on the nearest neighbor rule, which represents each RNA nucleotide by the SA letter whose corresponding exemplar nucleotide is nearest to the nucleotide being encoded. Finally, we applied BLAST to search all the SA-encoded sequences for RNA 3D substructures resembling the query RNA substructure.

**Table 1:** The structural alphabet of 23 conformational clusters with their associated capital letters and the *η* and *θ* pseudo-torsion angles of their center nucleotides

No.	Letter	(*η*,*θ*)	No.	Letter	(*η*,*θ*)	No.	Letter	(*η*,*θ*)
1	A	(161, 215)	9	I	(165, 8)	17	R	(67, 165)
2	B	(173, 243)	10	J	(181, 320)	18	S	(128, 212)
3	C	(201, 200)	11	K	(164, 267)	19	T	(78, 301)
4	D	(173, 166)	12	L	(231, 272)	20	V	(14, 344)
5	E	(200, 143)	13	M	(280, 232)	21	W	(304, 19)
6	F	(225, 108)	14	N	(327, 207)	22	Y	(337, 92)
7	G	(173, 97)	15	P	(4, 240)	23	Z	(256, 159)
8	H	(67, 79)	16	Q	(41, 228)			

**Fig. 3:**
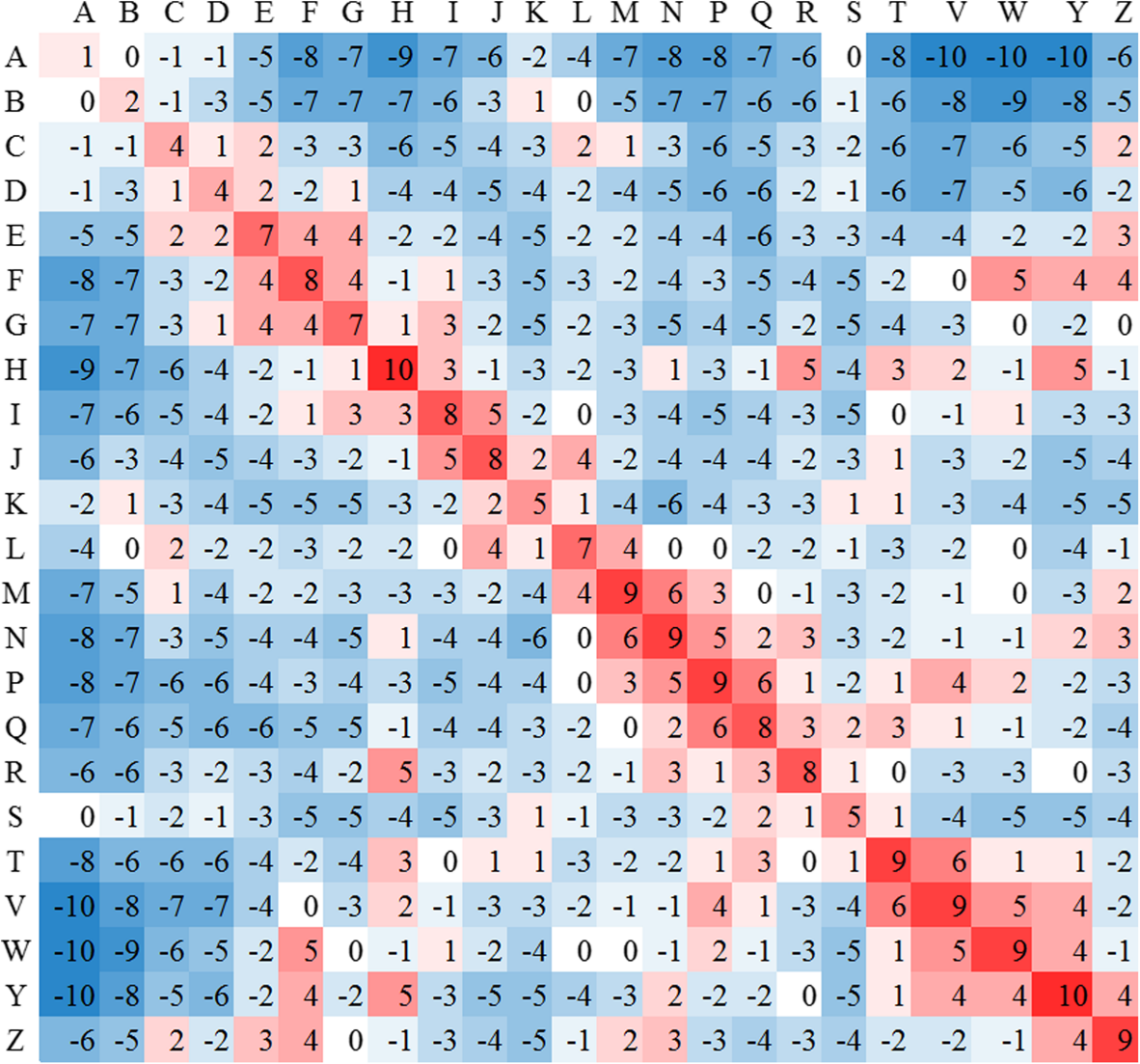
A BLOSUM-like substitution matrix used in R3D-BLAST2

For adequately scoring each alignment returned by R3D-BLAST2, we applied the statistical method proposed by Henikoff and Henikoff [16] on 237 equivalence classes with two or more RNA 3D structures in RNA 3D Hub [14] to derive a 23×23 BLOSUM-like substitution matrix (refer to Fig. 3). We also performed a search procedure in a grid style to optimize the gap open penalty by changing its value from − 15 to − 1 in steps of 1 and the gap extension penalty by changing its value from − 5 to − 0.5 in steps of 0.5. As a result, we chose − 12 and − 4 as the values of the gap open and extension penalties, respectively, in R3D-BLAST2. In addition, we computed the *E*-value of each R3D-BLAST2 search hit by using the equation *E*=*Kmn* ^−*λS*^ proposed by Karlin and Altschul [17], where *m* and *n* denote the sizes of the query RNA structure and the PDB database, respectively, *S* represents an alignment score between two SA-encoded sequences, and *K* and *λ* indicate statistical parameters relying on the search space size and the scoring function, respectively. Furthermore, we utilized the island method proposed by Altschul et al. [18] to obtain the estimated values of *K* and *λ* that are 0.072 and 0.4396, respectively.

Inevitably, R3D-BLAST2 may return some RNA 3D substructures that actually do not resemble any query RNA substructure. In fact, the *E*-values of these substructures are usually high. Therefore, we further equipped R3D-BLAST2 with an optional filter, which can screen out some returned RNA 3D substructures that do not pass user-defined thresholds of root mean square deviation (RMSD), structural alignment score (SAS) and/or percentage of structural identity (PSI), where SAS equals to 100×RMSD/(number of aligned residues) [19] and PSI is defined as a percentage of superimposed residues within 4.0 Å with respect to the length of the shorter of the two aligned structures [20]. For the sake of reducing running time, the above filter option in R3D-BLAST2 is not enabled by default.

### Usage of R3D-BLAST2

**Fig. 4:**
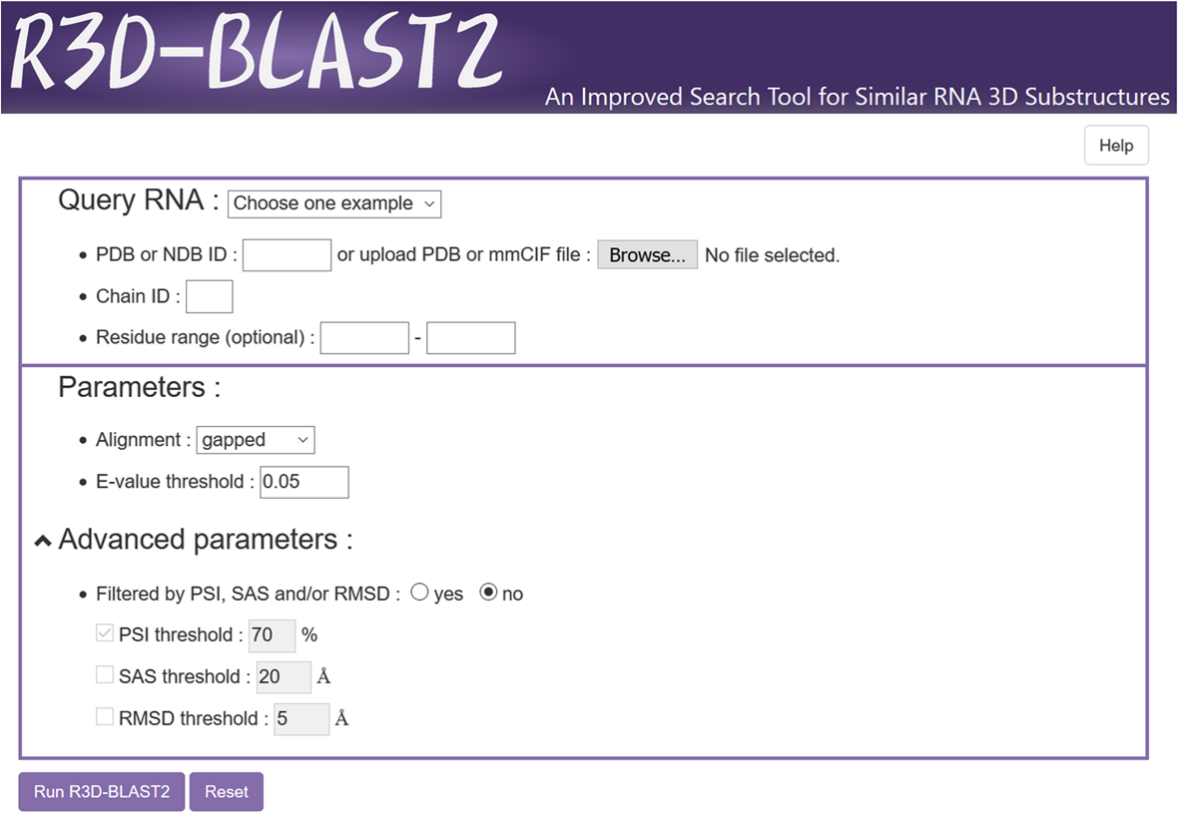
Interface of R3D-BLAST2

R3D-BLAST2 can be accessed at http://genome.cs.nthu.edu.tw/R3D-BLAST2/ by an easy-to-operate interface (see Fig. 4). It accepts a PDB/NDB ID or a PDB/mmCIF formatted file as a query RNA, along with a specified chain ID and optionally a specified residue range to be searched. If needed, the user can choose a specific alignment type, either gapped (default setting) or ungapped, to perform R3D-BLAST2 and change the predefined threshold of *E*-value to limit its search result. Furthermore, the user can screen the search result of R3D-BLAST2 by using the PSI, SAS and/or RMSD filters (whose thresholds are modifiable) in the ‘Advanced Parameters’ section. After that, R3D-BLAST2 quickly shows the details of each RNA substructure hit, such as PDB ID, chain ID, molecular function, structure determination method and structural resolution, as well as the information about its structural alignment against the query RNA, such as *E*-value, alignment range and coverage in both the query and subject RNAs, calculated PSI, SAS and RMSD (if the filter in the ‘Advanced Parameter’ section is selected) and a JSmol window displaying the superposition of aligned structures.

## Results and discussion

We utilized a benchmark dataset consisting of eight different kinds of RNAs (see Table 2) to test R3D-BLAST2 and also compared its performance to its previous version R3D-BLAST and other similar RNA structural search tools, such as RNA FRABASE, FASTR3D and RAG-3D. All the tools mentioned above were executed on their respective servers with default parameters unless specified otherwise.

**Table 2:** A benchmark dataset of eight RNA 3D structures

Query RNA	PDB ID	Chain ID	Length
Pseudoknot	2NOQ	B	46
Riboswitch	1Y27	X	68
Ribozyme	1VBY	B	73
tRNA	1EHZ	A	76
5S rRNA	3CC2	9	122
12S rRNA	5AJ3	A	960
16S rRNA	4ADV	A	1530
18S rRNA	3J80	2	1797

**Fig. 5:**
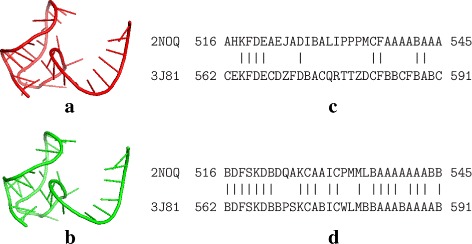
Two RNA substructures and alignments of their SA-encoded sequences: (**a**) a substructure of an RNA pseudoknot (PDB ID: 2NOQ, chain ID: B and residue range: 516–545), (**b**) a substructure of an 18S rRNA (PDB ID: 3J81, chain ID: 2 and residue range: 562–591), (**c**) an alignment with an identity of 30% between their SA-encoded sequences translated by the SA of R3D-BLAST, and (**d**) an alignment with an identity of 66.7% between their SA-encoded sequences translated by the SA of R3D-BLAST2

Consequently, as shown in Table 3, R3D-BLAST2 indeed found more RNA substructure hits that are similar to those of the query RNAs than its previous version R3D-BLAST. The main reasons for this result are as follows. First, as mentioned before, large RNA structures (e.g. ribosomal units) currently archived in the PDB database are only distributed in the mmCIF format and consequently they were missed by R3D-BLAST because of its incapability to retrieve RNA structures from the mmCIF formatted files. Second, more newly determined RNA structures were used to construct the SA of R3D-BLAST2 so that its SA letters have a higher capability to represent the most common backbone conformations of RNA nucleotides than those in the SA of R3D-BLAST. For example, given two substructures from two different RNAs as shown in Fig. 5, their SA-encoded sequences translated by the SA of R3D-BLAST2 share a much higher identity than those translated by the SA of R3D-BLAST, resulting in that the structural similarity between these two RNA substructures can be identified by R3D-BLAST2, but not by its previous version R3D-BLAST.

**Table 3:** Search results of RNA FRABASE (version 2), FASTR3D, R3D-BLAST, RAG-3D and R3D-BLAST2

Query RNA	R3D-BLAST2				R3D-BLAST	RAG-3D	RNA	FASTR3D
	100%	≥90%	< 90%	Total			FRABASE	
Pseudoknot	2	608	112	720 (1.0)	384 (1.0)	58 (1.9)	204 (0.7)	232 (0.7)
Riboswitch	11	45	20	65 (2.1)	53 (1.5)	10 (0.7)	1 (0.0)	1 (0.0)
Ribozyme	7	13	63	76 (1.2)	40 (1.8)	60 (0.2)	12 (0.5)	10 (0.4)
tRNA	78	397	1071	1468 (2.4)	560 (2.6)	10 (0.3)	59 (2.2)	19 (1.3)
5S rRNA	64	70	687	757 (2.2)	428 (2.0)	100 (0.1)	26 (0.3)	21 (0.3)
12S rRNA	2	2	7817	7819 (2.1)	3784 (2.0)	0 (0.0)	1 (0.0)	1 (0.0)
16S rRNA	1	132	4931	5063 (2.5)	2857 (2.7)	0 (0.0)	0 (0.0)	1 (0.0)
18S rRNA	1	7	8265	8272 (2.4)	4201 (2.2)	0 (0.0)	0 (0.0)	1 (0.0)

In the column titles of R3D-BLAST2, the percentage denotes the query coverage that equals to the percent of the query length in the alignment. The values presented in the parentheses indicate the average RMSD values (Å) between the structural hits and query, where notably the RMSD value returned by RAG-3D is calculated based on two superimposed RNA 3D graphs, rather than based on two superimposed RNA 3D structures as the other tools do

In addition, except pseudoknot and ribozyme in the benchmark dataset, R3D-BLAST2 still identified more RNA structure hits with 100% query coverage whose entire 3D structures are highly resemble the query RNA when comparing with RNA FRABASE and FASTR3D. Recall that RNA FRABASE and FASTR3D were both developed to search for RNAs that have the same 2D structure as the query RNA without any insertions and deletions. As demonstrated in our experimental results, therefore, they could inevitably miss those RNAs that possess the same overall 3D structures but different 2D structures and/or lengths. When queried with the pseudoknot and ribozyme in the benchmark dataset, both RNA FRABASE and FASTR3D returned more RNA structure hits as compared to the search result of R3D-BLASTS2 with 100% query coverage. In fact, some regions around these RNA 3D structures identified by RNA FRABASE or FASTR3D are not quite similar to the query RNA and as a result, they were removed from those RNA structure hits returned by R3D-BLAST2. In other words, all the pseudoknots and ribozymes returned by both RNA FRABASE and FASTR3D still can be found by R3D-BLAST2, but with a query coverage less than 100%. On the other hand, R3D-BLAST2 returned a large number of other structurally similar RNA substructures that actually are missed by both RNA FRABASE and FASTR3D.

**Fig. 6:**
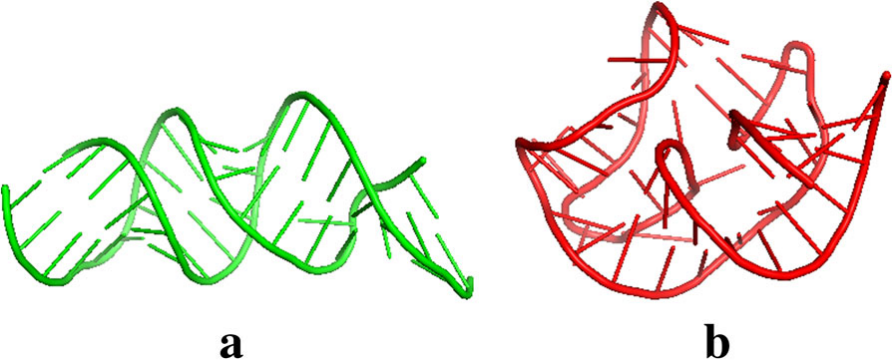
A substructure of an RNA (PDB ID: 4K27 and chain ID: U) (**a**) returned by RAG-3D when queried with an RNA pseudoknot (PDB ID: 2NOQ and chain ID: B) (**b**), where their graph-based RMSD calculated by RAG-3D is 2.565 Å

When compared to RAG-3D, R3D-BLAST2 was able to find many more RNA substructure hits that are quite structurally similar to the query RNA substructures. It should be noted that some of RNA substructures returned by RAG-3D may be structurally different from those of the query RNA, as shown in Fig. 6 for an example, even though their 3D graphs represented by RAG-3D are very similar to those of the query RNA. This is because the structural comparison performed by RAG-3D is a structural alignment between the 3D graphs of two RNA 3D structures and its RMSD calculated by RAG-3D is the so-called graph RMSD (rather than all-atom RMSD as computed by the other RNA structural search tools), which is used to measure the average distance of aligned vertices between two superimposed 3D graphs. In addition, RAG-3D seemed to be incapable of processing the queries of large RNA structures, such as 12S, 16S and 18S rRNAs.

**Fig. 7:**
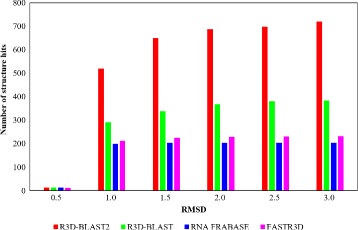
The accumulated number of RNA structure hits against their RMSD values reported by different RNA structural search tools when queried with the pseudoknot (PDB ID: 2NOQ and chain ID: B). Note that R3D-BLAST2, R3D-BLAST and RNA FRABASE all identified 13 RNA structure hits with RMSD ≤ 0.5 Å, while FASTR3D found 12 hits with RMSD ≤ 0.5 Å

Figure 7 displays the accumulated number of RNA structure hits against their RMSD values reported by different RNA structural search tools when queried with the pseudoknot in the benchmark dataset. Note that RAG-3D was excluded in this analysis because, as mentioned above, its RMSD is inconsistent with those reported by the other tools. Clearly, as shown in Fig. 7, R3D-BLAST2 was able to identify many highly similar RNA structure hits that were missed by the other tools.

Table 4 presents the running time of all the evaluated RNA structural search tools that was measured on their web servers when queried with each RNA 3D structure in the benchmark dataset. As shown in Table 4, each tool can finish its search job within a few to several tens of seconds, depending on the number of similar RNA structure and/or substructure hits and their coverages with respect to the query RNA.

**Table 4:** Comparison of running time (in seconds) for RNA FRABASE (version 2), FASTR3D, R3D-BLAST, RAG-3D and R3D-BLAST2

Query RNA	R3D-BLAST2	R3D-BLAST	RAG-3D	RNA FRABASE	FASTR3D
Pseudoknot	4.5	2.8	41.7	1.2	2.8
Riboswitch	0.8	0.5	2.9	1.0	0.3
Ribozyme	0.8	0.5	42.2	0.7	0.3
tRNA	11.9	2.3	2.7	1.9	0.3
5S rRNA	5.4	3.0	49.0	1.0	0.6
12S rRNA	29.6	17.6	12.4	6.5	0.7
16S rRNA	26.9	15.6	12.6	7.0	0.6
18S rRNA	38.5	23.0	28.2	4.4	1.0

## Conclusions

In this study, we upgraded our previous RNA structural search tool R3D-BLAST to develop a new web server named R3D-BLAST2 based on a newly constructed structural alphabet with a higher capability of representing the most common backbone conformations of RNA nucleotides. In contrast to the previous version, R3D-BLAST2 now can retrieve RNA 3D structures from the PDB data files in the mmCIF format and also allow the user to upload an mmCIF formatted file as an input to search for its similar RNA 3D substructures. According to our experimental results on a benchmark dataset, R3D-BLAST2 indeed outperforms its previous version R3D-BLAST and other similar RNA structural search tools RNA FRABASE, FASTR3D and RAG-3D by searching a larger collection of RNA 3D substructures resembling a query RNA substructure. It can be expected that R3D-BLAST2 will become a valuable tool to annotate RNA structures and functions because RNA molecules with the same function usually share similar 3D substructures.
